# How professional and academic pre-qualifications relate to success in medical education: Results of a multicentre study in Germany

**DOI:** 10.1371/journal.pone.0296982

**Published:** 2024-03-08

**Authors:** Carla Schröpel, Teresa Festl-Wietek, Anne Herrmann-Werner, Tim Wittenberg, Katrin Schüttpelz-Brauns, Andrea Heinzmann, Oliver Keis, Lena Listunova, Kevin Kunz, Tobias Böckers, Sabine C. Herpertz, Stephan Zipfel, Rebecca Erschens

**Affiliations:** 1 Department of Psychosomatic Medicine and Psychotherapy, Internal Medicine, University Medical Hospital Tuebingen, Tuebingen, Germany; 2 Medical Faculty, TIME -Tübingen Institute for Medical Education, University of Tuebingen, Tuebingen, Germany; 3 Department of General Psychiatry, Centre for Psychosocial Medicine, University of Heidelberg, Heidelberg, Germany; 4 Medical Education Research Department, Division for Study and Teaching Development, Medical Faculty Mannheim at Heidelberg University, Heidelberg, Germany; 5 Medical Faculty, Albert-Ludwigs-University Freiburg, Office of the Dean of Studies, Freiburg, Germany; 6 Medical Faculty, Office of the Dean of Studies, Ulm University, Ulm, Germany; 7 Medical Faculty Heidelberg, Heidelberg University, Deanery of Students’ Affairs, Heidelberg, Germany; 8 Deanery of Students’ Affairs, University’s Faculty of Medicine, Tuebingen, Germany; Ege University Faculty of Medicine, TURKEY

## Abstract

**Objective:**

Every year, many applicants want to study medicine. Appropriate selection procedures are needed to identify suitable candidates for the demanding curriculum. Although research on medical school admissions has shown good predictive validity for cognitive selection methods (undergraduate GPA, aptitude tests), the literature on applicants with professional and/or academic experience prior to entering medical school remains slim. In our study, we therefore aimed to examine the association between academic success in medical school and having previously completed vocational training in the medical field, voluntary service (≥11 months) or an academic degree.

**Methods:**

Data were collected in a multicentre, cross-sectional study at five medical schools in Germany (Baden-Wuerttemberg) from students during medical school (i.e. 3^rd^-, 6^th^-, and 10^th^-semester and final-year students). Academic success was assessed according to scores on the first and second state examinations, the total number of examinations repeated and the number of semesters beyond the standard period of study. For the analysis we calculated ordinal logistic regression models for each outcome variable of academic success.

**Results:**

A total of *N* = 2,370 participants (response rate: RR = 47%) participated in the study. Having completed vocational training was associated with a higher amount of repeated examinations (small effect), while having an academic degree was associated with worse scores on the second state examination (medium effect). No significant association emerged between voluntary service and academic success.

**Conclusion:**

The results indicate that professional and academic pre-qualifications pose no advantage for academic success. Possible associations with the financing of study and living conditions of students with pre-qualifications were analysed and discussed in an exploratory manner. However, the operationalisation of academic success from objective and cognitive data should be critically discussed, as the benefits of prior experience may be captured by personal qualities rather than examination results.

## Introduction

### Medical school admissions process

Professional and academic experience can shape people and influence how people think and act. Individuals who choose to pursue further education—for example, to study medicine after completing paramedic training—may begin that education with a different perspective and a broader range of experiences than ones without such prior experience. Prior practical experience may also be advantageous in getting admitted to medical school [1]. However, the literature addressing the role of professional and academic pre-qualifications in medical school admissions and their impact on medical students’ academic success remains slim. Nevertheless, research on appropriate criteria for selecting students to admit into medical school is important. On the one hand, the pool of applicants is large; on the other, the aim of medical schools is to admit students who are suitable for the medical profession [2].

Reviews on such selection methods have shown that overall undergraduate grade point average (GPA) and grades are significant predictors of academic success in medical school [3,4]. *Academic success* has been defined as referring to examination scores and other data regarding academic performance [5–8], as well as the number of examinations passed [9]. However, GPA alone as a criterion for selecting students for medical school becomes problematic when too many applicants with excellent grades apply [10]. Aptitude tests can measure cognitive skills other than qualifications earned upon exiting school [11] and help schools to select appropriate applicants. Of course, different aptitude tests are used in different countries. In the United Kingdom, the UK Aptitude Test or Biomedical Admissions Test are used to select medical students, and scores on both are associated with academic success [12,13]. In the United States and Canada, another aptitude test, the Medical School Admission Test, has also been shown in a meta-analysis to have low to moderate predictive validity for performance in medical school and the results of licencing examinations [14]. Meanwhile, in Germany, the Test for Medical Studies (TMS) used in medical school admissions has also been shown to significantly predict performance in medical school [5,15]. Even so, aptitude tests or school grades do not capture personal skills that may be important for studying medicine, including social skills, resilience and adaptability [16].

The literature shows that non-cognitive factors such as self-efficacy, effort and grade goal setting are positively associated with academic success [17–19]. However, there are few validated measures of non-cognitive factors that are actually used in the medical admissions process. For the valid assessment of non-cognitive data, Mini Mental Interviews (MMI) are a more effective method than unstructured interviews [3]. In MMIs, the non-cognitive attributes of applicants are recorded at different interview stations, similar to an objective structured clinical examination (OSCE) [20], and the interview process can be adapted to the specific medical school’s needs [21]. Other non-cognitive methods to assess personal qualities in the selection of medical students are Situational Judgement Tests (SJT) and tests assessing personality and emotional intelligence [3]. According to Patterson et al. [3], properly constructed SJTs are valid tools for medical selection, while the assessment of personality and emotional intelligence can be effective, but there may be a risk of faking or coaching. Internationally, medical schools continue to focus on cognitive performance in admissions rather than considering personal qualities [22]. Assessing prior qualifications may be an efficient way of identifying candidates who have already developed or tested their personal qualities through their previous experience.

### Professional pre-qualifications and academic success

In Germany, practical experience assessed using a points system can increase the likelihood of gaining a place at medical school. The selection process for medical studies in Germany follows centralised regulations and criteria. These regulations have been changed several times in recent decades [23]. Since 2020, medical schools have been required to allocate 10% of the available places on the basis of criteria independent of grades including vocational training, professional experience in the medical field, voluntary service, awards in education-related competitions, results of validated aptitude tests, situational judgement tests and interviews [24]. A further 30% of all study places are allocated based on the best undergraduate GPA and for overall 60% of all places, medical schools have been individually able to prioritise both criteria based on school leaving grades and criteria independent of grades via the “university selection procedure” (in German: “Auswahlverfahren der Hochschule, AdH”) [24]. However, previous practical experience has already been considered, both directly and indirectly, in the selection before 2020. On the one hand, the AdH has been in place since the winter semester 2005/2006. Since then, medical schools have been able to decide individually for 60% of the available places whether and how to weight criteria independent of school-leaving grades, such as vocational training, voluntary work, etc. For a detailed description on the AdH and medical school selection process in Germany see [25]. On the other, until 2020 (transition period until 2022) Germany applied the so-called “waiting list quota”. This quota could increase the chances of admission to medical school for people who had waited a certain number of semesters for admission. From winter semester 2005/2006 onwards, 20% of all available study places were allocated according to this quota. During their waiting time, these applicants had often already completed a related vocational training (e.g. nursing, paramedics) [26].

To date, few studies have examined the relationship between prior professional or academic qualifications and academic success. In a review, Erschens et al. [25] summarised studies that examined how completing vocational training influences academic success. Although studies on the topic were too few to draw any clear conclusions, the authors did identify a trend, at least in the preclinical part of the study, of a slight disadvantage among students with vocational training in terms of data concerning academic performance and the number of examinations passed [9,27]. However, other evidence suggests the positive impact of prior vocational training on academic success. In a recent study, Amelung et al. [5] found that vocational training positively affected performance on all examinations in the first two years of study. They thus suggested that such medical students may have acquired important skills during their training that led to better performance—for example, higher levels of stress resistance and medical knowledge. Still other evidence indicates that medical, nursing and physician associate’s-degree students with previous experience in healthcare may have a clearer idea of the professional roles of healthcare professionals and therefore a better understanding of the needs and concerns of the different members on medical teams [28].

Similar to having completed vocational training, earning an academic degree indicates having already completed a full education. In admissions for medical study in Germany, applicants with a second degree are considered for a preliminary quota, along with other quotas for foreigners and cases of hardship [29]. Nevertheless, no research has produced results showing how studying before entering medical school affects the course of medical study. On a smaller scale, people who have completed voluntary service, similar to ones who have completed vocational training, have already been able to gain practical experience before attending medical school. In Germany, voluntary service of at least 11 months, including social and ecological service, is considered in the allocation of places for medical study. However, when Hampe et al. [9] examined that particular subgroup, they found no significant advantage of academic performance for medical students who had completed voluntary service before their studies.

### Integration of findings

In our study, we focused on applicants who had already gained practical experience over a longer period or acquired knowledge or competence through study. Having completed an academic degree, voluntary service or vocational training in the medical field is summarised in our study as “professional and academic pre-qualifications”. On the one hand, applicants with professional and academic pre-qualifications have already acquired practical knowledge (e.g. in vocational training or voluntary service) or theoretical knowledge (e.g. in university), which may make it easier for them to study medicine. On the other, medical study is further education for applicants with prior vocational training or an academic degree. For this subgroup, some time has passed since they graduated from school and due to changed life circumstances (part-time job, family) other priorities than getting the best grades might be important. Furthermore, the experience of voluntary service may not be sufficient to influence performance in medical studies due to the short duration of one year.

However, because research examining how professional and academic pre-qualifications impact success in medical school remains slim [25], we sought to investigate this association in our study. To examine that relationship as broadly as possible we collected data from medical students in different semesters (i.e. 3^rd^-, 6^th^-, and 10^th^-semester and final-year students) at five medical schools using different outcome variables to assess academic success. The aim of our study was:

- To analyse the associations between professional and academic pre-qualifications (i.e. voluntary service, academic degree and vocational training in the medical field) and academic success of medical students (i.e. scores on the 1^st^ and 2^nd^ state examinations, the total number of examinations repeated and the number of semesters beyond the standard period of study).

## Materials and methods

### Design and sample

This cross-sectional, multicentric study was conducted at five medical schools in Germany. For the present study, at each school, medical students at different stages of their medical education—that is, students in their 3^rd^, 6^th^ or 10^th^ semester or in their final year of study—were invited to participate via email or recruited during lectures.

### Setting

**Fig 1:**
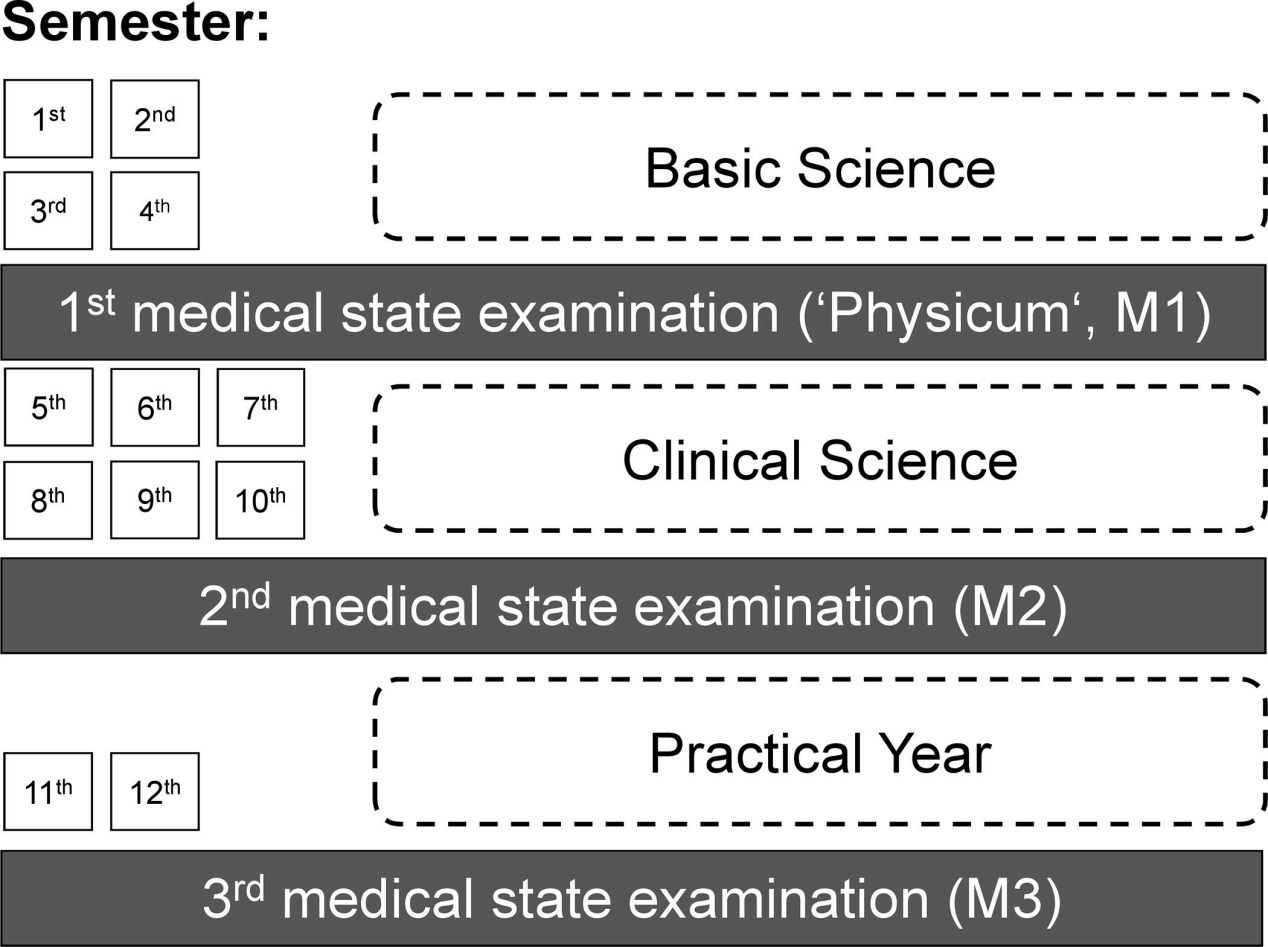
Illustration of the structure of the medical curriculum with regard to the timing of the three major examinations in Germany (state examinations M1-M3) and semester levels. This figure is an adaption from Erschens [30].

The standard duration of medical study in Germany is 12 semesters (i.e. 6 years). Along with the regular examinations in each semester, medical students need to pass three major examinations (i.e. state examinations M1–M3). Completing the so-called final year and passing the M3 marks the end of medical education and permits newly qualified doctors to begin additional specialty training. Fig 1 is a figure adapted from Erschens [30] and illustrates the different semester levels and timing of the state examinations.

### Measures and procedure

The study was conducted as part of a collaborative research project titled “Pre-qualifications for Human Medicine” focused on the relationship between pre-qualifications, academic success and other study-related co-variables. The data presented here represent the main -analysis of this project, focusing on variables that assess academic success. The overall questionnaire for the project included items addressing participants’ socio-demographic background (e.g. age, living situation and parents who work in the medical field), admissions to medical school, how they were financing their studies and other study-related aspects. Sub-questions on secondary outcome variables including professional burnout, student satisfaction, preferences for speciality training and learning strategies will be published elsewhere. These sub-questions can be clearly distinguished from each other. The data collection took place between the winter semester 2019/2020 (beginning 1^st^ of October) and the summer semester 2021 (30^th^ of September). Data were collected from all relevant semesters at each of the five medical schools. The exact start and end dates of the survey varied between medical schools and semesters for organisational reasons (e.g. different timetables, different dates for presenting the study during lectures). At the end of the survey period, medical students in the same semester were grouped together, regardless of when they completed the questionnaire. The survey was anonymous, and participants provided their informed consent (written or online) to participate before completing the online or paper-and-pencil questionnaire.

#### Academic and professional pre-qualifications

In a team of experts, we developed a questionnaire addressing professional and academic pre-qualifications, including items on vocational training, studies and voluntary service prior to medical study. For all three types of pre-qualifications, questions inquired into the area in which the activity conferring the qualification was gained, whether the activity was completed, the final grade when applicable and, in the case of an academic degree or vocational training, whether the activity was followed by work in the same area of training or study. Because practical experience (e.g. voluntary work and vocational training) is and has been considered in admissions to medical school in Germany (the emphasis and weighting varies between medical schools), another question inquired into whether the activity was started in order to increase the likelihood of gaining a place at medical school. See S1 Table in the appendix for the full questionnaire. Based on the admissions criteria for medical school in Germany [24], we defined *vocational training* as completed professional training in the medical field. To create the variable vocational training (yes, completed vocational training in the medical field vs. no), only vocational training that was completed with a grade and that was completed in the medical field was included. Also based on the German admission criteria for medical school, *voluntary service* was defined as volunteer work lasting at least 11 months (yes, completed voluntary service of at least 11 months vs. no). As a voluntary service of less than 11 months is not considered for admission to medical school in Germany, it was therefore not included in the definition. Last, we defined an *academic degree* as a degree completed with a grade and made no distinction between bachelor’s and master’s degrees or the areas of study (yes, prior academic degree vs. no). We chose this definition because in Germany the preliminary quota allows second degree students to be considered regardless of the subject studied.

#### Academic success

**Fig 2:**
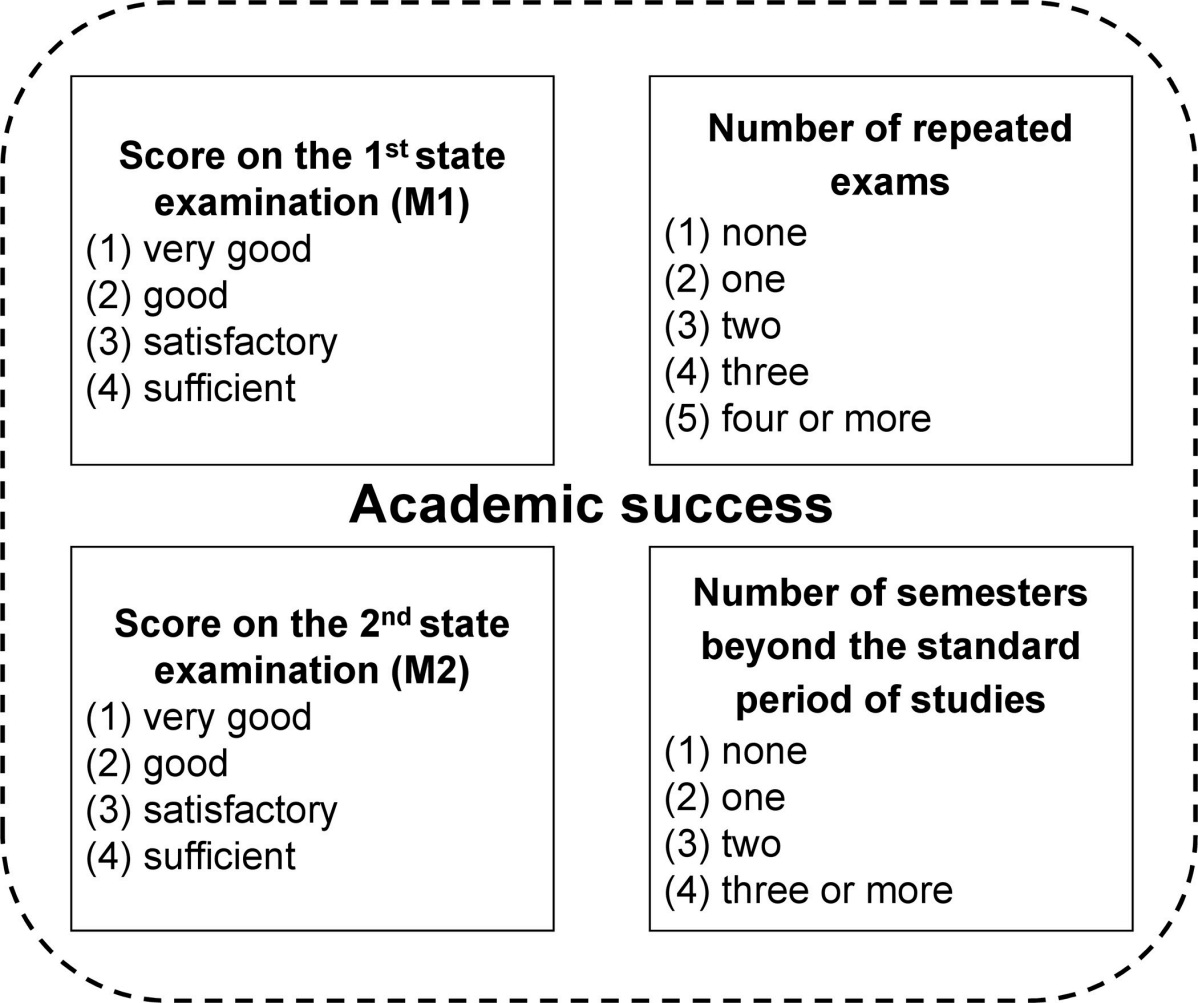
This figure illustrates the four dependent variables used in our study to assess academic success. Each variable has an ordinal response format, with higher numbers indicating lower academic success.

To measure academic success, we examined four ordinal variables, shown in Fig 2. Due to centralised examination dates in Germany, grades for the second state examination are only available in later semesters.

### Ethics

The study was approved by the Ethics Committees of the Faculty of Medicine at Tübingen University Hospital (No. 534/2019BO1). All methods were carried out according to the Declaration of Helsinki Association (2013). Participation in the study was voluntary and participants gave their informed consent (written or online). Book vouchers were distributed to participants as compensation.

### Statistical analysis

For all analyses, we used SPSS version 28.0.0.0 (IBM, Armonk, NY, USA), and percentages were rounded to the nearest whole number. Given the cross-sectional design of the study, we did not perform a power analysis prior to the study. Due to the multicentre structure of the data, a multilevel approach with ordinal outcome was chosen for the analysis; the distribution function was multinomial, while the link was cumulative log odds. In an intercept-only model we tested for significant variations across the five different medical schools, and if no level 2 differences were found, then single-level models were conducted. For single-level ordinal regression analysis, we checked for multicollinearity and the proportional odds assumption; the higher the category of the four outcome variables, the worse the academic success. Meanwhile, the predictor variables were vocational training, academic degree and voluntary service (yes = 0, no = 1). For missing values, the corresponding case was excluded, and the level of significance was set at α < .05. We also calculated odds ratios (ORs) to determine the effect size, which we interpreted following Chen et al. [31] according to values of approximately 1.5 (small effect), approximately 3.0 or more (medium effect), and approximately 5.0 (large effect).

#### Control variables

We added gender (female = 0, male = 1) and undergraduate GPA (in German, ‘Abitur’, in which lower numbers indicate of better grades) to all four models. In the model to predict grades on the M2, we added score on the M1 as a predictor variable. In the models to predict the number of repeated examinations and the number of semesters beyond the standard period of study, we added a control variable, level of experience with medical school, as a metric predictor (i.e. levels 0–11, with each semester corresponding to one level); it was added to control for the potential accumulation of repeated examinations and semesters beyond the standard period of study as the number of semesters increased.

## Results

### Response rate and sample description

Altogether, *N* = 2,370 medical students (i.e. in their 3^rd^, 6^th^, or 10^th^ semester or final year), participated in the study. The response rate was about 47%. As part of the survey was conducted online, we cannot confirm whether the respondents actually received the email with the invitation. If the response rate would have been calculated based on the number of people who actually read the email rather than the number of email addresses, it would probably be higher. It is possible that some recipients may not have opened the email, e.g. due to illness, parental leave, a semester abroad or similar. However, for technical reasons, we do not know the number of emails opened.

Most medical students were female (*n* = 1521, 65%); participants’ ages ranged between 18 and 45 years (*M* = 24.40, *SD* = 3.78). On average, medical students reported an undergraduate GPA of *M* = 1.48 (*SD* = 0.53). Descriptively, medical students in different semesters reported similar proportions of having completed vocational training in the medical field, with the highest percentage in the 6^th^ semester (30%) and the lowest percentage in the final year (23%). The proportions of medical students who reported having completed an academic degree prior to medical school were also similar across semesters, with the highest percentage in the 6^th^ semester (7%) and the lowest percentage in the final year (5%). Also, the proportions of medical students reporting having completed a voluntary service were similar across semesters, with the highest percentage in the 6^th^ semester (26%) and the lowest percentage in the final year (19%). Table 1 provides an overview of the distinct characteristics of the different semester levels in the sample.

**Table 1:** Overview on the characteristics of the different semester levels in the sample.

		semester				total sample
		3^rd^	6^th^	10^th^	final year	
***N***	***n*(%)**	635(27)	729(31)	485(20)	521(22)	2370 (100)
***age [years]***	***M*(*SD*)**	22.17(3.58)	23.98(3.41)	25.66(3.34)	26.51(3.24)	24.40 (3.78)
	***range***	[18,43]	[19,45]	[21,41]	[19, 42]	[18, 45]
**gender**						
**female**	***n*(%)**	413(65)	464(64)	312(65)	332(64)	1521(65)
**male**	***n*(%)**	218(34)	255(35)	167(35)	186(36)	826(35)
**diverse**	***n*(%)**	1(<1)	2(<1)	0(0)	1(<1)	4 (<1)
**undergraduate GPA** ^**1**^	***M(SD)***	1.48(0.54)	1.49(0.51)	1.48(0.54)	1.48(0.52)	1.48(0.53)
	***range***	[1.00,3.40]	[0.80,3.70]	[0.70,3.50]	[1.00,3.30]	[0.70,3.70]
**professional and academic pre-qualifications**						
**vocational training**	***n*(%)**	185(29)	218(30)	132(27)	120(23)	655(28)
**academic degree**	***n*(%)**	37(6)	53(7)	28(6)	27(5)	145(6)
**voluntary service**	***n*(%)**	161(25)	189(26)	97(20)	99(19)	546(23)

*Note*. Percentages rounded to the nearest whole number.

^1^undergraduate grade point average, in “German Abitur”, lower numbers stand for better grades.

### Professional and academic pre-qualifications

**Fig 3:**
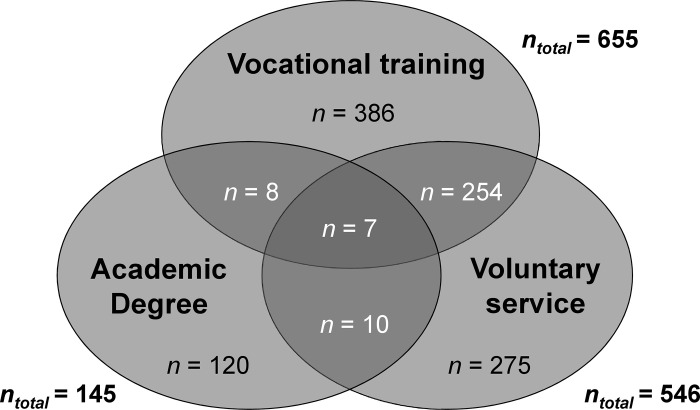
This figure illustrates the number of reported single, double and triple pre-qualifications. Overlaps are presented in a darker colour. The number of students who completed all three qualifications before medical school is shown in the centre of the three circles.

Overall, *N* = 1,060 participants (45%) reported having completed at least one of the three professional and academic pre-qualifications. The greatest overlap surfaced among participants who had completed vocational training and voluntary service; of all individuals who had completed vocational training, approximately 40% also completed a voluntary service. Fig 3 shows the numbers of participants with single, double and triple qualifications. Approximately 28% (*n* = 655) of all medical students reported having completed vocational training in the medical field prior to their studies. Training as a paramedic (43%, *n* = 283) or nurse (36%, *n* = 238) was most commonly reported, and most reported having completed vocational training to increase their chance of being admitted to medical school (67%, *n* = 435). Meanwhile, approximately 6% (*n* = 145) reported having an academic degree. Most students had a degree in a STEM subject (30%, *n* = 44; i.e. science, technology, engineering and mathematics) or in the medical field (28%, *n* = 41; i.e. dentistry, molecular medicine and human biology). Approximately 23% (*n* = 546) of all students reported engaging in voluntary service. Most had volunteered in the health sector (40%, *n* = 215) or in a sector not listed as an option on the questionnaire (42%, *n* = 229). A detailed description of the professional and academic pre-qualifications appears in Table 2.

**Table 2:** A detailed description of the type, extent and other characteristics of professional and academic pre-qualifications in the sample.

	total sample	
	*n*	*%*
**vocational training in the medical field**		
yes	655	28
no	1707	72
area		
paramedic	283	43
nurse	238	36
health sector^1^	128	20
pharmaceutical sector	4	1
geriatric care	2	<1
remedial care	0	0
completed training to increase chances of being admitted to medical school		
yes	435	67
no	214	33
final grade of the training		
***M*(*SD*)**	1.62(0.53)	
**range**	[1.00,3.60]	
work experience in years		
***M*(*SD*)**	2.99(2.53)	
**range**	[0.20,19.00]	
**university degree**		
yes	145	6
no	2214	94
area		
STEM^2^	44	30
medical field^3^	41	28
field of psychology	14	10
nursing and health sciences	5	3
pedagogy and educational sciences	3	2
home economics or nutritional sciences	3	2
pharmacy or pharmaceutical technology	1	1
social work, social education	1	1
other	33	23
final grade		
***M*(*SD*)**	1.54(0.45)	
**range**	[1.00,2.80]	
**voluntary service (≥ 11 months)**		
yes	546	23
no	1813	77
area		
health care	215	40
child and youth welfare	55	10
care for the disabled	28	5
care of the elderly	16	3
welfare	1	<1
in another field	229	42
voluntary service to increase chances of being admitted to medical school		
yes	257	47
no	288	53

*Note*. n.a. = not available; percentages rounded to the nearest whole number.

^1^including speech and language therapists, physiotherapists and medical assistant

^2^including mathematics, computing, biology, chemistry or physics

^3^including dentistry, molecular medicine, human biology.

### Professional and academic pre-qualifications and academic success

The intercept did not vary significantly across the five different medical schools for any outcome variables: score on the M1 (*z* = 1.28, *p* = .201), score on the M2 (*z* = 0.84, *p* = .401), number of repeated examinations (*z* = 1.31, *p* = .189) and number of semesters beyond the standard period of study (*z* = 1.31, *p* = .191). Thus, we conducted single-level models without controlling for medical school. The model’s assumptions concerning proportional odds were met for all ordinal regression models, and no multicollinearity emerged between the independent variables with a variance inflation (VIF) factor between 1.01 and 1.81 [32]. All of the results of the ordinal regression are shown in Table 3.

**Table 3:** Results of the ordinal logistic regression models for the four variables measuring academic success.

**Score on the M1,** χ^2^(5) = 230.82, *p* < .001;							
*n* = 1656, Mc Fadden *R*^2^ = .06							**Score on the M1:** very good = 24%, good = 45%, satisfactory = 25%, sufficient = 6%
	***ß***	***SE*(*ß*)**	***p***	***OR***	**95%CI(*OR*)**		
predictors							
vocational training	0.24	0.13	.070	1.27	[0.98,	1.65]	
academic degree	0.22	0.19	.260	1.24	[0.85,	1.82]	
voluntary service	0.03	0.11	.810	1.03	[0.82,	1.29]	
undergraduate GPA	1.26	0.12	< .001***	3.54	[2.80,	4.47]	
gender	0.70	0.10	< .001***	2.01	[1.65,	2.45]	
**Score on the M2,** χ^2^(6) = 182.84, *p* < .001							
*n* = 409, Mc Fadden *R*^2^ = .22							**Score on the M2:** very good = 2%, good = 49%, satisfactory = 41%, sufficient = 8%
	***ß***	***SE*(*ß*)**	***p***	***OR***	**95%CI(*OR*)**		
predictors							
vocational training	0.16	0.30	.599	1.17	[0.65,	2.09]	
academic degree	1.19	0.43	.005**	3.29	[1.42,	7.59]	
voluntary service	0.14	0.27	.595	1.15	[0.68,	1.95]	
M1 grades	1.46	0.15	< .001***	4.31	[3.20,	5.81]	
undergraduate GPA	0.77	0.27	.004**	2.15	[1.28,	3.63]	
gender	-0.09	0.22	.671	0.91	[0.59,	1.41]	
**Number of repeated exams,** χ^2^(6) = 272.98, *p* < .001							
*n* = 2289, Mc Fadden *R*^2^ = .06							**Number of repeated exams:** none = 66%, one = 16%, two = 8%, three = 5%, ≥ four = 5%
	***ß***	***SE*(*ß*)**	***p***	***OR***	**95%CI(*OR*)**		
predictors							
vocational training	0.47	0.12	< .001***	1.61	[1.27,	2.03]	
academic degree	-0.12	0.19	0.539	0.89	[0.61,	1.29]	
voluntary service	0.05	0.11	0.631	1.05	[0.86,	1.29]	
undergraduate GPA	0.97	0.10	< .001***	2.63	[2.16,	3.21]	
gender	0.30	0.10	.002**	1.34	[1.11,	1.62]	
Level of experience	0.10	0.01	< .001***	1.11	[1.08,	1.14]	
**Number of semesters beyond the standard period of studies,** χ^2^(6) = 878.12, *p* < .001							
*n* = 2294, Mc Fadden *R*^2^ = .19							**Number of semesters beyond:** none = 63%, one = 18%, two = 15%, ≥ three = 4%;
	***ß***	***SE*(*ß*)**	***p***	***OR***	**95%CI(*OR*)**		
predictors							
vocational training	-0.23	0.14	.092	0.79	[0.60,	1.04]	
academic degree	-0.24	0.21	.254	0.79	[0.52,	1.19]	
voluntary service	-0.05	0.12	.694	0.95	[0.75,	1.21]	
undergraduate GPA	0.14	0.12	.247	1.14	[0.91,	1.44]	
gender	0.43	0.10	< .001***	1.54	[1.25,	1.88]	
Level of experience	0.47	0.02	< .001***	1.60	[1.54,	1.66]	

*Note*. For all dependent variables, the highest category was taken as reference category—higher categories indicate poorer academic performance; percentages rounded to the nearest whole number. The reference category for vocational training was “no vocational training”, for academic degree it was “no academic degree” and for voluntary service it was “no voluntary service”. The reference category for the control variable gender was “male”.

* *p* < .05

** *p* < .01

*** *p* < .001.

None of the professional and academic pre-qualifications contributed significantly to the model with score on the M1 as dependent variable. However, both control variables contributed significantly to the model. The odds of having higher scores on the M1 increased with increasing undergraduate GPA (*OR* = 3.54). For female students, the odds of having higher scores on the M1 were higher than for male students (*OR* = 2.01).

The odds of having a higher score on the M2 were higher for students with than without an academic degree (medium effect, *OR* = 3.29); however, no effect emerged for having completed vocational training or voluntary service. All control variables except for the variable gender contributed significantly to the model. The odds of having a higher score on the M2 increased with increasing undergraduate GPA (*OR* = 2.15) and increasing M1 grades (*OR* = 4.31).

For students with prior vocational training, the odds of reporting more repeated examinations were higher than for students without prior vocational training (small effect, *OR* = 1.61); however, no effect surfaced for a prior academic degree or completed voluntary service. All control variables contributed significantly to the model. The odds reporting more repeated examinations were higher for female than for male students (*OR* = 1.34). With increasing undergraduate GPA (*OR* = 2.63) and level of experience (*OR* = 1.11), the odds of reporting more repeated examinations increased.

None of the professional and academic pre-qualifications contributed significantly to the model with the number of semesters beyond the standard period of study as the dependent variable. All control variables except for undergraduate GPA contributed significantly to the model. For female students, the odds of reporting a higher number of semesters beyond the standard period of studies were higher than for male students (*OR* = 1.54). With increasing level of experience the odds of reporting a higher number of semesters beyond the standard period of studies increased (*OR* = 1.60).

All significant *p* values would have survived Bonferroni correction for multiple testing (α_adj_ = α/4).

### Exploratory data analysis

In an exploratory analysis, associations between professional or academic pre-qualifications and selected socio-demographic characteristics were examined. The purpose was to provide ideas for future research on why pre-qualifications may not be an advantage for academic success. We calculated χ^2^-Tests to analyse the association between socio-demographic data and pre-qualifications. To test for significant differences in the mean undergraduate GPA, we calculated Welch *t*-tests. All results can be found in Table 4. Below we describe the associations that have become significant.

**Table 4:** Marital status, children, parents who work in the medical field, undergraduate GPA and financing of studies by professional and academic pre- qualifications.

		vocational training		*p*	academic degree		*p*	voluntary service		*p*
		yes	no		yes	no		yes	no	
**Marital status and children:**										
marital status				< .001			.004			.278
living in a partnership	***n*(%)**	403(60)	728(44)		84(60)	1042(47)		271(50)	854(48)	
not living in a partnership	***n*(%)**	265(40)	944(56)		56(40)	1155(53)		269(50)	943(52)	
own children (living in own household)				< .001			.813			.721
yes	***n*(%)**	66(10)	24(1)		6(4)	83(4)		22(4)	67(4)	
no	***n*(%)**	609(90)	1662(99)		139(96)	2130(96)		524(96)	1745(96)	
**Parental education:**										
at least one parent with a degree in medicine or dentistry				< .01			.979			.024
yes	***n*(%)**	124(18)	420(25)		33(23)	506(23)		106(19)	436(24)	
no	***n*(%)**	552(82)	1266(75)		112(77)	1708(77)		440(81)	1377(76)	
at least one parent with a university degree in another subject				< .001			.463			.413
yes	***n*(%)**	329(49)	1010(60)		78(54)	1260(57)		318(58)	1020(56)	
no	***n*(%)**	347(51)	676(40)		67(46)	954(43)		228(42)	793(44)	
no parent with tertiary education				< .001			.443			.395
yes	***n*(%)**	258(38)	374(22)		43(30)	592(27)		154(28)	478(26)	
no	***n*(%)**	418(62)	1312(78)		102(70)	1622(73)		392(72)	1335(74)	
parents who work in the medical field				< .01			.396			.998
one parent	***n*(%)**	169(25)	322(19)		36(25)	453(20)		113(21)	376(21)	
both parents	***n*(%)**	74(11)	239(14)		16(11)	294(13)		72(13)	240(13)	
no parent	***n*(%)**	430(64)	1125(67)		93(64)	1464(66)		361(66)	1194(66)	
**GPA**:										
undergraduate GPA^1^	***M(SD)***	1.96(0.56)	1.29(0.36)	< .001	1.62(0.52)	1.47(0.52)	< .001	1.64(0.54)	1.43(0.51)	< .001
	***range***	[1.00,3.50]	[0.70,3.70]		[0.90,3.10]	[0.70,3.70]		[1.00,3.50]	[0.70,3. 70]	
graduation (undergraduate GPA)				< .001			.117			< .001
in Germany	***n*(%)**	660(98)	1525(91)		128(90)	2052(94)		540(99)	1641(91)	
other country	***n*(%)**	13(2)	142(9)		14(10)	142(6)		3(1)	153(9)	
**Financing studies by:**										
parents/relatives										
yes	***n*(%)**	448(66)	1544(92)	< .001	112(77)	1876(85)	.016	445(82)	1543(85)	.042
no	***n*(%)**	228(34)	142(8)		33(23)	338(15)		101(18)	270(15)	
partner/spouse										
yes	***n*(%)**	81(12)	41(2)	< .001	12(8)	110(5)	.081	37(7)	84(5)	.047
no	***n*(%)**	595(88)	1645(98)		133(92)	2104(95)		509(93)	1729(95)	
BaföG^2^										
yes	***n*(%)**	127(19)	136(8)	< .001	4(3)	258(12)	< .001	72(13)	190(10)	.078
no	***n*(%)**	549(81)	1550(92)		141(97)	1956(88)		474(87)	1623(90)	
own earnings										
yes	***n*(%)**	521(77)	963(57)	< .001	102(70)	1381(62)	.054	392(72)	1090(60)	< .001
no	***n*(%)**	155(23)	723(43)		43(30)	833(38)		154(28)	723(40)	
loan (e.g. student loan)										
yes	***n*(%)**	39(6)	45(3)	< .01	10(7)	74(3)	.025	21(4)	63(3)	.682
no	***n*(%)**	637(94)	1641(97)		135(93)	2140(97)		525(96)	1750(97)	
scholarship										
yes	***n*(%)**	86(13)	371(22)	< .001	18(12)	437(20)	.030	83(15)	373(21)	< .01
no	***n*(%)**	590(87)	1315(78)		127(88)	1777(80)		463(85)	1440(79)	
savings										
yes	***n*(%)**	307(45)	394(23)	< .001	51(35)	647(29)	.128	215(39)	485(27)	< .001
no	***n*(%)**	369(55)	1292(77)		94(65)	1567(71)		331(61)	1328(73)	
other										
yes	***n*(%)**	22(3)	50(3)	.993	13(9)	59(3)	na^3^	14(3)	57(3)	.487
no	***n*(%)**	654(97)	1636(97)		132(91)	2155(97)		532(97)	1756(97)	

*Note*. Percentages rounded to the nearest whole number.

^1^undergraduate grade point average = “Abitur” in Germany, low numbers stand for better grades.

^2^”Bundesausbildungsförderungsgesetz” is a common German student loan, funded by the state.

^3^na = no χ^2^-test could be calculated due to expected cell frequencies <5.

The proportion of medical students living in a partnership (*p* < .001), having own children who live in the same household (*p* < .001), or having no parent with tertiary education (*p* < .001) was higher among those with vocational training than among those without. The proportion of students with at least one parent with a degree in medicine or dentistry (*p* < .01) or at least one parent with a university degree in another subject (*p* < .001) was lower among those with vocational training than among those without. There was also a significant association between vocational training and having parents who work in the medical field (*p* < .01). Meanwhile, the proportion of students who financed their studies with the help of a partner or spouse (*p* < .001), BaföG (p < .001, German financial aid programme funded by the state) a loan (*p* < .01), their own earnings (*p* < .001) or savings (*p* < .001) was also higher among those who had completed vocational training than among those without. The proportion of students who reported financing their studies with the help of parents or relatives (*p* < .001), or a scholarship (*p* < .001) was lower among those with vocational training than among those without.

The proportion of students who reported financing their studies with the help of parents or relatives (*p* = .016), BaföG (*p* < .001), or a scholarship (*p* = .030) was lower among those with an academic degree than among those without. Meanwhile, the proportion of medical students who reported living in a partnership (*p* = .004) and financing their studies with a loan (*p* < .01) was higher among those with an academic degree than among those without.

The proportion of medical students who reported financing their studies with the help of a partner or spouse (*p* = .047) or through their own earnings (*p* < .001), and savings (*p* < .001), was higher among those with voluntary service than among those without. However, the proportion of students with at least one parent with a degree in medicine or dentistry (*p* = .024) and who reported financing their studies with a scholarship (*p* < .01) was lower among those with voluntary service than among those without.

For those with vocational training (*p* < .001), as well as for those with voluntary service(*p* < .001) or an academic degree (*p* < .001), the undergraduate GPA was on average worse than for those with no prior qualifications. Also, the proportion of medical students who graduated from school in Germany was higher among those with vocational training (*p* < .001) or voluntary service than among those without.

## Discussion

In our study we aimed to investigate the relationship between professional and academic pre-qualifications and academic success for medical students in their 3^rd^, 6^th^ or 10^th^ semester or their final year (*N* = 2,370). Data were collected in a cross-sectional, multicentre study at five medical schools in Germany (Baden-Wuerttemberg). Academic success was operationalised by the scores obtained on the first and second state examination (i.e. M1 and M2), as well as by the number of repeated examinations and semesters beyond the standard period of study.

Having completed vocational training was associated with a higher number of repeated examinations (small effect); however, no such association emerged with score on the M1 or M2 or with the number of semesters beyond the standard period of study. Although having an academic degree before entering medical school was associated with worse scores on the M2 (medium effect), no such association occurred with score on the M1, the number of semesters beyond the standard period of study or the number of repeated examinations. Voluntary service before medical study was not associated with academic success. In an exploratory analysis, especially for medical students who completed vocational training before entering medical school, we found associations with responsibility for own children and type of student financing. The results of the exploratory data analysis are included in the following discussion of the findings.

### Professional pre-qualifications and academic success

Similar to Hampe et al. [9], we found no association between volunteering before medical school and academic success. Although voluntary service provides important practical experience that may contribute to personal development, its duration may be too brief to impact success in medical school. In our exploratory analysis we found that individuals who had volunteered prior to medical school tended to report different financing of their studies. For example, the proportion of students who financed their studies through their own earnings and savings was higher among students with prior volunteering experience than among those without. However, students were able to indicate several ways of financing their studies (multiple choice), and it is not clear from our data what proportion of their studies are self-financed. In addition, the exploratory analysis showed that the undergraduate GPA of students who had completed voluntary service was worse than that of the group without voluntary service. This may be an indication of why this group could not start studying medicine directly after school. Overall, the proportion of students with at least one parent with a degree in medicine or dentistry was lower among those with voluntary service than among those without. These findings may indicate that the interest or decision to study medicine may not have come from role models at home, but from personal experiences during voluntary service, which the majority of our sample undertook in the health sector.

To the best of our knowledge, no research has produced results showing how studying before entering medical school affects success in medical school. We did not find any association between variables of academic success and having an academic degree except for score on the M2. Because the second state examination is usually taken after the tenth semester, individuals who have studied another subject before studying medicine have studied for at least eight years. That group, due to other life circumstances, may have other priorities than achieving top scores on the M2. However, our exploratory analysis did not provide a clear picture of what these circumstances might be. Overall, however, more students with an academic degree reported living in a partnership. We could not find an association between having own children and an academic degree. At least the proportion of those who reported financing their studies with the help of parents or relatives, a scholarship or BaföG (German state-funded financial aid) was lower in the group with an academic degree than in the group without. However, the association between having an academic degree and lower scores on the M2 could also be mainly due to worse school leaving grades. In our exploratory analysis, the undergraduate GPA was worse for those with an academic degree than for those without. Further studies should examine other factors that may explain the association with worse scores on the M2.

We are not aware of any literature review, other than the review of Erschens et al. [25], that specifically examines individuals with both practical and academic experience prior to attending medical school. In particular, the authors investigated the associations of pre-qualifications with objective data, non-cognitive data and preference for specialty training. However, due to the small number of studies available on the topic, a trend could be identified for the objective data only. Erschens and colleagues concluded that students with vocational training tended to perform below average in the first years of study.

The findings of our study align with the results Hampe et al. [9,27], who also found no advantage for medical students who had completed vocational training. The operationalisation of academic success chosen by Hampe et al. [9] was similar to ours, and they found a lower number of passed examinations among students who had completed vocational training. Our exploratory analysis showed that students with vocational training often reported financing their studies with their own earnings, which suggests a possible additional burden and less time for studying due to having to work part-time jobs. Furthermore, the proportion of medical students with children living in the same household, who financed their studies with savings, with the help of a partner or spouse or with a (student) loan was higher among those with vocational training than among those without. The results of our exploratory analysis suggest that individuals with vocational training may differ from individuals without vocational training in terms of additional burden factors. These may be factors that influence the amount of repeated examinations. Beyond that, practical experience may not be able to compensate for the intensive memorisation required for written examinations. Among other results, we found no association between the number of semesters beyond the standard period of study and prior vocational training. Presumably, repeated examinations are compensated for by additional work so that the standard period of study can be adhered to. In addition, our exploratory analysis showed that the proportion of medical students with completed vocational training in the medical field, who reported not having a parent with a tertiary education, was higher than for those without vocational training. They were also less likely to report that at least one parent had a degree in medicine, dentistry or another subject. As those with vocational training also reported lower undergraduate GPA, this may indicate a link between parents’ educational background and children’s educational opportunities. In their article, Becker and Hecken [33] discuss why working-class children are less likely to take the direct path to university after leaving school. Further studies should investigate this association in medical students.

However, there is evidence that medical students’ practical experience may have a subjective effect on their performance in medical school [34]. Our results contrast with the findings of Amelung et al. [5], who found a positive influence of vocational training on the mean percentile rank over all performance data points in the first two years of study. However, we operationalised success by scores on the M1 and M2 and by the number of repeated examinations and semesters beyond the standard period of study. The different operationalisations of academic success not only complicate comparisons of the studies but also show the importance of discussing the concept of academic success, which according to Patterson et al. [3] is controversial in research on admissions in which performance outcomes should be predicted (i.e. a criterion problem). In medical school, other achievements may be more important than later in practice. Therefore, it is impossible to conclude from our data who will become a good doctor. By contrast, Simmenroth-Nayda et al. [35] and Heidmann et al. [36] examined neither grades nor other exam-related results but the results of MMIs. Both studies revealed that medical students with vocational training performed slightly better on a MMI and in an applicant interview than other students. The MMI consisted of different stations with medical situations such that the applicants’ reactions and social skills could be recorded. When it comes to practical tasks, people with vocational training may outperform ones without such training. Thus, additional studies should explore the concept of academic success by covering it as broadly as possible and, whenever possible, including relevant soft skills in the assessment. A study by Tsikas and Afshar [37] who provides some initial insight into alternative examinations in medical school in association with pre-qualifications. They examined the relationship between work experience before entering medical school and scores on a clinical examination after the second year of medical school. The practical clinical examination (OSCE) consisted of nine stations covering the categories "physical examination", "medical skills", and "communication". The authors found, that medical students with prior practical experience scored higher on practical medical skills (e.g. drawing blood), but not on communication skills.

### Professional pre-qualifications in the admissions process

Overall, we found a very high proportion of medical students in our sample with professional or academic pre-qualifications. A total of 28% reported vocational training, 6% an academic degree, and 23% voluntary service. Overall, it should be noted that these percentages are self-reported and do not necessarily reflect the objective percentages at German medical schools.

One possible explanation for the high proportions in voluntary service and vocational training could be, among other things, the German admission system. Until 2022 Germany applied the so-called "waiting list quota". This quota could increase the chances of admission to medical school for people who had waited a certain number of semesters for admission. It is possible that these individuals began vocational training or volunteer work during the waiting period and were able to begin medical school afterward. In addition, for some years now, a quota system in Germany has placed special emphasis on prior practical experience. In this context, medical schools could decide whether and how to weight certain criteria that are independent of grades. As a result, practical experience can increase the chances of being admitted to medical school.

However, our high percentages are in line with the results of DeWitt and Harrison [1]. The authors reported that 36% of all accepted applicants in their sample reported scribing experience in a healthcare setting. In the USA scribes help physicians with the documentation. Erschens et al. [25], who provide an overview of the frequency of previous vocational qualifications reported in studies in their review, reported heterogeneous rates ranging from 15% to 86.7%. Those results may be due to the varying emphasis that universities place on previous experience in the admissions process.

We could not find an advantage of professional and academic pre-qualifications for academic success. However, we were able to replicate the effect of undergraduate GPA on academic success [3,4] which we used as a control variable. The better the GPA the better the grades on the M1 and M2 and the lower the number of repeated examinations. In contrast, we did not find an association between undergraduate GPA and the number of semesters behind the standard period of study. Prior undergraduate performance still appears to be an important predictor of academic success. However, there are recommendations from selection research to consider personal qualities when selecting appropriate medical students [22]. It would also be conceivable to include non-academic attributes in the selection process that characterise good doctors [38]. Asking about previous professional or academic experience could be an efficient way of including personal qualities in the selection process.

Another advantage beyond the practical knowledge of the applicant group with professional or academic pre-qualifications could be a higher motivation to study and become doctors. Wouters et al. [39] found that medical students who experienced a more elaborate admissions process reported higher post-admission motivation than students who obtained placement due to undergraduate GPA or lottery. Applicants not directly admitted due to undergraduate GPA and who first engage in vocational training, voluntary service or studies may have to wait longer and invest more to be admitted to medical school. Studies have also shown that academic self-efficacy [17–19] and effort [19], which can be counted as motivational beliefs and behaviours [40], are positively related to academic success. In addition, grade goal setting can be associated with academic success [18]. However, in our study, prior practical experience did not lead to better academic performance. It may be that people with previous practical experience have higher self-efficacy in relation to practical tasks and patient contact. Motivational constructs such as effort and self-efficacy in relation to academic performance may be more pronounced in the group of those who already had good grades at school, regardless of previous academic and practical pre-qualifications. Further studies should investigate these non-cognitive variables in relation to prior practical or academic experience.

It is striking that approximately 40% of participants who had completed vocational training in the medical field in our study also reported having completed voluntary service. Possible explanations are that the group had to wait a long time for placement in medical school and decided to train in the meantime and that voluntary service motivated them to work in the medical field and convinced them to engage in training. Last, it should be noted that not only should medical students have the prerequisites to become good students and good doctors, but the curriculum is constantly being modified to best prepare students for the medical profession.

### Strengths and limitations

A particular strength of our study was that we examined a very large sample of medical students in different semesters, with data collected at five medical schools in Germany. Our results make an important contribution to a topic that has been little explored. Moreover, a wide range of variables was chosen to operationalise success, including not only the results of two state examinations but also adherence to the standard duration of study and the number of repeated examinations. However, our definition of academic success lacks an outcome that explicitly captures non-cognitive but practical skills. Further studies should examine professional and academic pre-qualifications e.g. in relation to results in MMI or OSCE. Within our study team we considered combining OSCE results with our data, but due to data restrictions (such as data privacy issues) this was not possible. Another limitation is that our cross-sectional study precludes investigating the progress of individuals over their course of study. As some medical students have completed more than one pre-qualification, these cannot be considered completely independent of each other. In addition, our data is not objective, but self-reported. Our operationalisation of completed voluntary service, previous vocational training in the medical field and previous academic degree as dummy variables excludes medical students who dropped out of voluntary service, vocational training or academic studies before completion. Another limitation is that the dummy-coding of previous professional and academic qualifications did not include information about the duration of any subsequent work experience. The duration of training, which is generally three years in Germany, was also not recorded. In Germany there is a training programme for paramedical assistants, which is often followed by work experience, but is much shorter. As we have not explicitly excluded this short programme, it is possible that some of the people who have indicated “paramedic” as their vocational training have only completed a short training programme. Future studies could examine the relationship between work experience and academic success in order to assess the importance of having many years of work experience. For such studies, we recommend also collecting qualitative data to better understand the importance of work experience.

### Conclusion

We found no advantage of prior professional or academic qualifications for academic success in medical school. Nevertheless, it remains unclear how to best operationalise academic success and whether such success relates to professional success after graduation. Research on admissions can benefit from our findings, which derive from the assessment of the presence of different prior professional and academic experiences in relation to academic success. Admissions procedures should take into account that prior professional and academic experience may have had a beneficial effect on personal qualities, thereby enabling them to enter medical school with an advantageous perspective and experience that eventually cannot be captured by the results of examinations.

## Supporting information

S1 Table (DOCX)
