# Supplementary material for: How professional and academic pre-qualifications relate to success in medical education: Results of a multicentre study in Germany
Source: PLoS One. 2024 Mar 8;19(3):e0296982. doi: 10.1371/journal.pone.0296982 (PMC10923489; doi:10.1371/journal.pone.0296982)
Supplement: S1 Table — (DOCX) [file pone.0296982.s001.docx]

**Supporting Information**

**S1 Table. Questionnaire on professional and academic pre-qualifications, including items on vocational training, studies and voluntary service prior to medical school, translated from German**

| **Vocational training** | |
| --- | --- |
| Did you start or complete any vocational training before starting medical school? | Yes |
|  | No |
| If yes, which type of training did you start or complete? | ... in nursing |
|  | ... in geriatric care |
|  | ... in remedial care |
|  | ... as a paramedic |
|  | ... in another health profession/health sector (e.g. speech and language therapy, physiotherapy, medical assistant) |
|  | ... in the pharmaceutical sector |
|  | ... in a technical field (e.g. automotive engineering, electrical professions) |
|  | ... in the field of creative work |
|  | … in the craft sector |
|  | … in another sector |
| The training was completed with the following overall grade: | [grade] |
| Did you start your training to increase the likelihood of being admitted to medical school? | Yes |
|  | No |
| After you finished your vocational training, did you work in your trained profession? If yes, please indicate the duration (in years): | [years] |
| **Academic degree** | |
| Have you started or completed any other academic studies before medical school? | Yes |
|  | No |
| If yes, which study programme did you start or complete? | ... in the medical field (e.g. dentistry, molecular medicine  human biology) |
|  | ... in the field of nursing or health sciences |
|  | ... in the field of pharmacy or pharmaceutical technology |
|  | ... in STEM disciplines (e.g. mathematics, computing, biology, chemistry or physics) |
|  | ... pedagogy and educational sciences |
|  | ... in the field of psychology |
|  | ... in the field of social work or social education |
|  | ... in the field of home economics or nutritional sciences |
|  | ... in another field |
| You have completed your studies with the following overall grade: | [grade] |
| After you finished your study programme, did you work in the field of your studies? If yes, please indicate the duration (in years): | [years] |
| **Voluntary service** | |
| Did you do any voluntary service before medical school? | Yes |
|  | No |
| If yes, in which area did you complete your voluntary service? | ... health care |
|  | … care for the elderly |
|  | ... care for the disabled |
|  | ... child and youth welfare |
|  | ... welfare |
|  | … in another field |
| The duration of the voluntary service (in months): | [months] |
| Did you do the voluntary service to increase the likelihood of being admitted to medical school? | Yes |
|  | No |
